# Numerical Study on the Effects of Thermo-Electromagnetic Force on Solute Transport and Microstructural Evolution in a Directionally Solidified Al-2.5 wt.% Cu Alloy

**DOI:** 10.3390/ma19112267

**Published:** 2026-05-27

**Authors:** Fengli Ren, Zhicong Ding, Gang Wang, Ming Yang, Xiaofeng Xu, Honghao Ge

**Affiliations:** 1 College of Optical, Mechanical and Electrical Engineering, Zhejiang A&F University, Hangzhou 311300, China; rfl@zafu.edu.cn (F.R.); 13962236324@163.com (Z.D.); xxf@zafu.edu.cn (X.X.); 2 School of Safety Science and Engineering, Henan Polytechnic University, Jiaozuo 454000, China; yming@hpu.edu.cn; 3 Zhejiang Universe Filter Co., Ltd., Wenzhou 325204, China; wggg525@126.com; 4 College of Mechanical Engineering, Zhejiang University of Technology, Hangzhou 310014, China

**Keywords:** grain growth, solute distribution, steady magnetic field, microstructure, cellular automaton method

## Abstract

Thermo-electromagnetic force plays a crucial role in tailoring the solidification microstructure by altering thermal-solutal buoyancy. However, while in situ synchrotron experiments offer some observations of microstructural evolution, their restricted spatial resolution and beam intensity prevent the full characterization of fluid flow and solute transport during solidification. To address this limitation, a calibrated model of a cellular automaton method coupled with a Eulerian multiphase approach is employed in this study to comprehensively investigate the impact of solute distribution on grain evolution during the directional solidification of an Al-2.5 wt.% Cu alloy under varying steady magnetic fields from 0.5 T to 4.0 T. The model incorporates heat and solute transport, nucleation, grain growth, and complex melt flows driven by thermal-solutal buoyancy, alongside thermo-electromagnetic effects and induced Lorentz forces. Simulations reveal that under a steady 0.5 T magnetic field, an elliptical copper-rich region forms near the solidification front. This solute redistribution significantly influences the development of a tilted solid–liquid interface, consistent with experimental observations. As the magnetic field strength increases, this copper-rich region transitions from an elliptical to a circular morphology. Notably, under a 4.0 T magnetic field, the tilted interface is effectively stabilized due to the suppression of grain growth. Furthermore, significant grain refinement is observed under a steady magnetic field, as the average grain size decreases from 209.3 μm without magnetic field to 122.5 μm of 0.5 T. This refinement is driven by redistribution of the copper concentration, which increases the undercooling from 1.4 K to 3.7 K and generates new nucleation zones. This solute-driven mechanism is identified as the primary cause of grain refinement under steady magnetic fields and is successfully validated by experimental results. These results shed new light on the mechanism of grain growth evolution under a steady magnetic field.

## 1. Introduction

Solidification is a fundamental process in a foundry. However, traditional solidification techniques are often insufficient for effectively controlling solute segregation and defect formation during grain growth. Over the past few decades, magnetic field-assisted solidification [1,2,3] has emerged as a vital technology for regulating grain size and mitigating segregation, primarily through the manipulation of melt flow and thermal-solutal buoyancy. During magnetic field-assisted solidification, potential differences induced by temperature gradients at the solid–liquid interface generate a thermo-electromagnetic force (TEMF) [4]. Compared with traditional solidification, the induced TEMF significantly alters melt convection, modifying heat and mass transport in ways that ultimately dictate the final microstructure. Therefore, a comprehensive investigation into the regulatory mechanisms of TEMF on grain growth and solute distribution holds profound scientific significance and engineering value.

A few experimental studies have been carried out to reveal the regulatory mechanisms of a steady magnetic field to tailor the microstructure [5,6,7], nucleation [8], and segregation [9]. Recently, in situ synchrotron radiography has been employed for the direct observation of microstructural evolution [10,11]. However, capturing the dynamic details of melt flow and solute transport remains challenging due to limitations in spatial resolution and beam intensity, making numerical simulation an essential complementary tool to bridge this observational gap. At the macroscale, various multi-physics models [7,12] have been developed to investigate the evolution of melt flow patterns driven by TEMF during solidification, and the numerical results are in good agreement with the experimental results. Conversely, at the microscale, simulations incorporating the effects of TEMF on grain growth remain scarce due to the prohibitive computational costs associated with such complex models.

However, driven by rapid advancements in computational power, researchers have recently begun developing numerical models to simulate microstructure evolution under the influence of TEMF. For instance, a multi-physics phase–field–lattice Boltzmann model has been developed to simulate the columnar grain growth under an external magnetic field [13], and the simulation results reveal that thermo-electromagnetic convection is induced near the solid–liquid interface, accompanied by the formation of vortices between dendritic arms. However, the computational domains in their study are typically restricted to length scales on the order of 100 μm. This is significantly smaller than actual experimental dimensions, thereby precluding direct quantitative comparisons. Furthermore, a parallelized numerical code coupling alloy solidification with fluid flow, driven by both buoyancy and Lorentz forces, was developed to reveal the mechanisms by which magnetohydrodynamics alters the solidification of microstructures [11]. These numerical findings were directly compared with high-speed synchrotron X-ray imaging results. However, the temperature field within the computational domain is prescribed a priori, and grain nucleation is not taken into account in their model. Recently, a three-dimensional cellular automaton method coupled with a Eulerian multiphase approach has been developed to investigate dendritic growth under a steady magnetic field by our group [14]. Although our previous model successfully reproduced the formation of a tilted solid–liquid interface, the restricted scale of the computational domain prevented a comprehensive understanding of solute transport, grain nucleation, and the mechanisms underlying grain refinement.

In this study, a cellular automaton method coupled with a Eulerian multiphase approach is developed and calibrated to systematically investigate the impact of solute distribution on grain evolution during the directional solidification under various steady magnetic fields. Because the perfect eutectic reaction occurs in the Al–Cu alloy with a lower concentration of copper, an Al-2.5 wt.% Cu alloy is adopted in the current study. Crucially, the computational domain is extended to the millimeter scale compared with our previous micrometer scale, which fits the experimental sample dimensions of millimeter scale. Thereby, a more comprehensive phenomena of melt flow and heat and mass transfer will be presented in current study. The model rigorously accounts for heat and solute transport, grain nucleation and growth, complex melt flows driven by thermal-solutal buoyancy, and TEMF effects and the induced Lorentz forces. Through this framework, the formation of a tilted solid–liquid interface, along with solute redistribution, microstructural evolution, and the underlying grain refinement mechanisms, are thoroughly elucidated.

## 2. Materials and Methods

### 2.1. Model Assumptions

The current model was developed on the basis of our previously established numerical frameworks, and detailed descriptions can be found in the relevant literature [15,16]. Here, only a brief overview of the present numerical model is provided. The model accounts for melt flow induced by thermal-solutal buoyancy and TEMF, as well as heat and mass transfer phenomena during solidification. For each phase, the temperature, phase fraction, solute concentration, and liquid flow field are obtained by solving the corresponding governing equations, which are summarized in Table 1. The fundamental assumptions adopted in the present model are as follows:

- Two phases are considered, namely the liquid (l) and solid (s) phases.
- All thermophysical properties are assumed to be constant, because the simulation focuses on the melt’s solidification with a relative limit of decreasing temperature.
- Because of the low Reynolds number in the current situation, laminar flow is considered and the liquid phase is treated as a movable Newtonian fluid, whereas the solid phase is assumed to be stationary.
- Solidification shrinkage, dendrite deformation, and movement are neglected.

### 2.2. Mass Transfer

The mass transfer between the liquid and solid phases is defined as

(1) $$M_{\mathrm{ls}}=\frac{\Delta f_{s}\cdot \rho_{l}}{\Delta t}$$

where $\Delta f_{s}$ is the change in the solid fraction, $\rho_{l}$ is the liquid density, and Δt is time step. The increment in the solid fraction is defined as

(2) $$\Delta f_{s}=\frac{c_{l}^{*}-c_{l}}{c_{l}^{*}(1-k)}\cdot G$$

where $c_{l}^{*}$ is the equilibrium solute concentration at the solid–liquid interface, $c_{l}$ is the local solute concentration, *k* is the solute partition coefficient, and $G=\min[\frac{1}{2}(\sum_{m=1}^{4}S_{m}^{a}+\frac{1}{\sqrt{2}}\sum_{m=1}^{4}S_{m}^{b}),1]$ is a geometrical factor that depends on the state of neighboring cells [17].

### 2.3. Momentum Transfer

The velocity field of the liquid phase is obtained by solving the momentum conservation equation. The drag force between the liquid and solid phases is modeled using the Kozeny–Carman permeability law [18], which is expressed as

(3) $$K_{s}=\frac{d^{2}f_{l}^{3}}{180f_{s}^{2}}$$

Meanwhile, the Boussinesq approach is used to model the thermal and solutal buoyancy

(4) $${\vec{F}}_{\rho }=f_{l}\rho_{l}\vec{g_{l}}$$

where $\vec{g_{l}}={[\beta }_{T}\cdot \left(T^{\mathrm{ref}}-T_{l}\right)+\beta_{c}\cdot (c^{\mathrm{ref}}-c_{l})]\cdot \vec{g}$, $\beta_{T}$ is the thermal expansion coefficient, $\beta_{c}$ is the solutal expansion coefficient, and $\vec{g}$ is the gravity vector.

In addition, an electric current is induced along the solid–liquid interface, forming a closed-loop circulation due to differences in thermoelectric potential. This current is expressed as

(5) $$\vec{J}=\sigma (\vec{E}-S\nabla T+\vec{u}\times \vec{B})$$

where σ is the electrical conductivity, $\vec{E}$ is the electric field, *S* is the Seebeck coefficient, $\vec{u}$ is the melt velocity, and $\vec{B}$ is the magnetic field.

### 2.4. Energy Transfer

The local temperature is obtained by solving the corresponding enthalpy conservation of two phases and the heat exchange is defined as

(6) $$Q_{\mathrm{ls}}=Q_{\mathrm{ls}}^{p}+Q_{\mathrm{ls}}^{d}$$

where $Q_{\mathrm{ls}}^{p}=\left\{\begin{array}{c} {h_{l}\cdot M}_{\mathrm{ls}}\;solidification\\ h_{s}\cdot M_{\mathrm{ls}}\;remelting \end{array}\right.$ and $Q_{\mathrm{ls}}^{d}=H^{*}\cdot (T_{l}-T_{s})$, where H* is an artificial volumetric heat transfer coefficient introduced to balance the local enthalpy between the liquid and solid phases to acquire one temperature in the grids, which is 1.0 × 10^9^ W/m^2^/K.

### 2.5. Species Transfer

The solute concentrations for two phases are calculated from the corresponding species equations, and the solute exchange term is defined as

(7) $$C_{\mathrm{ls}}=\left\{\begin{array}{c} {k\cdot c_{l}^{*}\cdot M}_{\mathrm{ls}}\;solidification\\ c_{s}\cdot M_{\mathrm{ls}}\;remelting \end{array}\right.$$

where *c*_s_ is the species concentration of the solid phase, and the species diffusion is considered in both the solid and liquid phases.

### 2.6. Nucleation Model

The nucleation conditions have an important influence on the microstructure during solidification processing. A Gaussian distribution [19] can be applied to characterize the nucleation density as a function of undercooling

(8) $$n(\Delta T)=\frac{n_{\max}}{\sqrt{2\pi }\Delta T_{\sigma }}\int_{0}^{\Delta T}\exp[-\frac{1}{2}(\frac{\Delta T-T_{N}}{\Delta T_{\sigma }})]d(\Delta T)$$

where *n*_max_ is the maximum density of the grains, $\Delta T_{\sigma }$ is the standard deviation of undercooling, and $\Delta T_{N}$ is the average value of nucleation undercooling.

### 2.7. Simulation Setting

**Figure 1:**
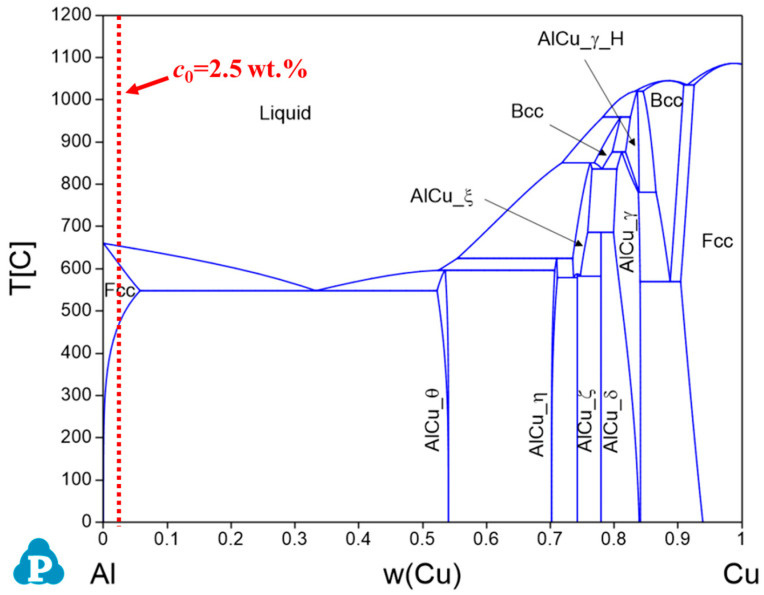
Phase diagram [21] for the Al–Cu alloy.

**Figure 2:**
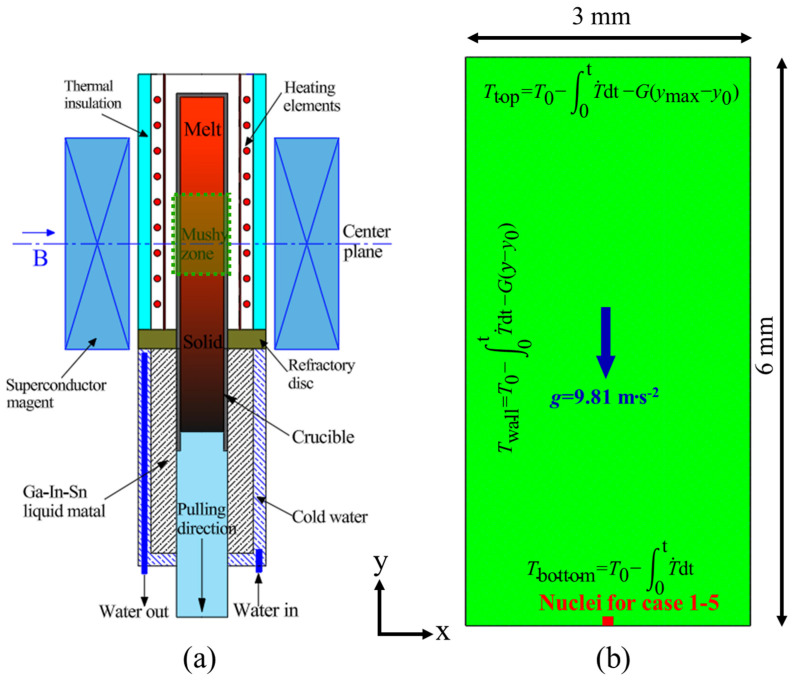
The schematic description of (**a**) the experimental setup and (**b**) the initial and boundary conditions for the simulation cases.

The detailed phase diagram for the Al-Cu alloy is presented in Figure 1, and the initial concentration of copper is 2.5 wt.% as marked as a red line. The conservation equations for current simulation cases are summarized in Table 1, and the physical properties of Al-2.5 wt.% Cu alloy employed in all cases are presented in Table 2. Additionally, the processing parameters of pulling rate and thermal gradient used in current research are summarized in Table 3, which were used to fabricate the experimental samples presented in the literature [20] by the experiment setup shown in Figure 2a. All equations are solved by a phase coupled SIMPLE (PC-SIMPLE) algorithm. The time step is determined by $\Delta t\leq \frac{{\Delta x}^{2}}{4.5D_{l}}$, and for every time step, 60 iterations are necessary to keep convergence during the numerical calculation, and the detailed numerical parameters used in all simulation cases are shown in Table 4. Here we want to emphasize that the mushy zone is focused as a simulation domain under a transverse magnetic field during the directional solidification, and the simulation domain is 3000 μm × 6000 μm with a grid size of 20 μm, smaller than the primary dendritic space of about 200 μm. For the columnar grain cases, named as cases 1–5, a nuclei is pre-set at the center of the bottom area and grows as the temperature progresses. However, for the cases of equiaxed grain, the nucleation model will be activated to calculate the density of the grains as the temperature progresses. Meanwhile, for all simulation cases, the initial and boundary conditions are the same and presented in Figure 2b.

## 3. Results

### 3.1. Model Validation

**Figure 3:**
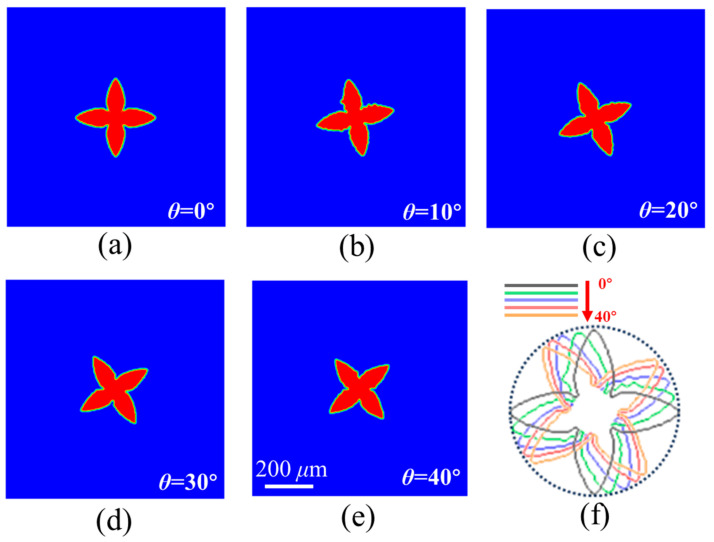
Morphologies of dendritic grains with different preferred orientations: (**a**) 0°, (**b**) 10°, (**c**) 20°, (**d**) 30°, and (**e**) 40°. (**f**) Qualitative comparison of the outlines.

**Figure 4:**
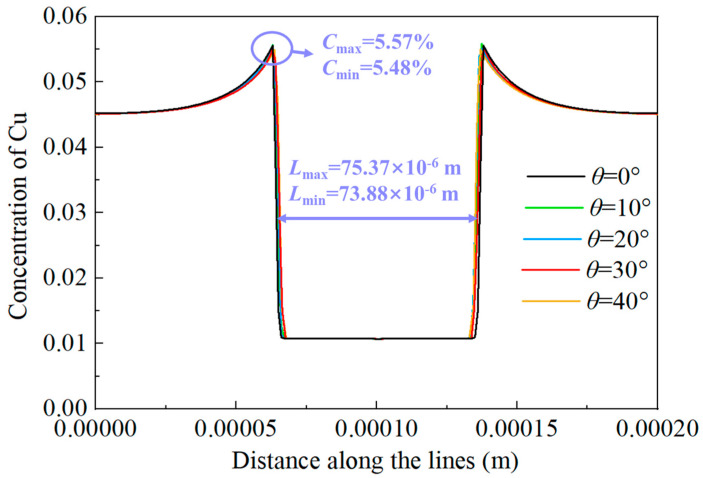
Comparison of dendrites’ front solute concentration and dendrite lengths with different orientations.

During solidification simulation, the cellular automaton method is prone to grid anisotropy, which causes differences in grain growth rates. Specifically, the grain tends to grow along the 0° and 45° directions in an orthogonal grid. Therefore, it is necessary to perform grid independence verification when simulating the grain growth with different initial orientations. For the validation simulation, five cases with initial angles of 0°, 10°, 20°, 30°, and 40° were conducted with the preferential orientation at the center of simulation domain because of the quadruple symmetry in the current morphology of grains. During solidification, the nuclei grow and form a fourfold symmetric dendritic structure. The lengths of the dendrites with different preferential orientations are measured after 0.08 s, and the simulation results are shown in Figure 3. Detailed morphologies of dendrites with different preferential orientations are presented in Figure 3a–e. Additionally, the outlines for each dendrite with different preferential orientations are extracted to be compared in Figure 3f. After qualitative comparison, all simulation results indicate that the dendrites maintain their preferential growth orientations and exhibit similar lengths after 0.08 s. Furthermore, by analyzing the concentration field distribution at the front of each dendrite, the lengths of dendrites with different orientations can be quantitatively assessed, as shown in Figure 4. The maximum copper concentration at the dendritic tips with five different orientations is quite close, with a deviation of 1.6% from the maximum value of 5.57 wt.% to the minimum value of 5.48 wt.%. Meanwhile, the dendritic lengths of these five different orientations are with a deviation of 2.0% from the maximum value of 75.37 × 10^−6^ m to the minimum value of 73.88 × 10^−6^ m. All in all, the simulation results demonstrate that the dendrites can maintain their inherent preferential orientation during solidification without any effects from grid anisotropy, which also shows that this cellular automaton model is relatively reasonable, with a deviation of 1.6% with different initial grain orientations.

### 3.2. Effects of a Steady Magnetic Field

**Figure 5:**
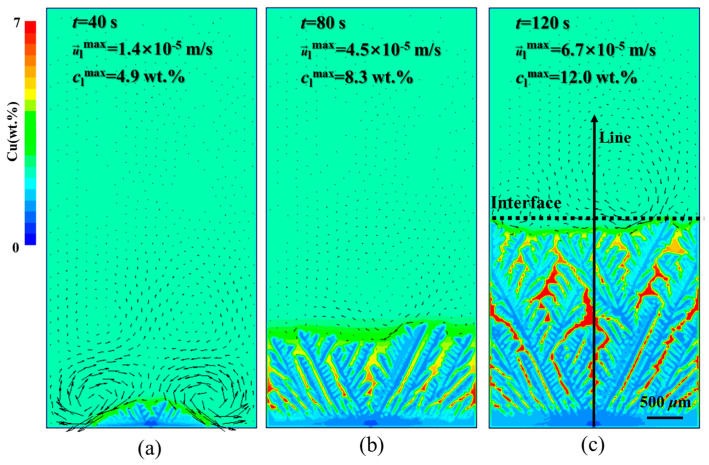
Typical characterization of columnar grain evolution, melt flow state, and solute distribution without any magnetic effect: (**a**) 40 s, (**b**) 80 s, and (**c**) 120 s.

The case without the effects of a steady magnetic field (Case 1) is simulated, and the typical evolutions of the grain morphology sequence, melt flow state, and distribution of copper concentration are presented in Figure 5. Due to the effect of gravity, two primary buoyancy-driven forces are considered to govern the melt flow: (a) thermal buoyancy, associated with a positive thermal expansion coefficient, which drives the downward flow of the high-temperature melt; and (b) solutal buoyancy, associated with a negative solutal expansion coefficient, which induces the downward flow of the copper-enriched melt. Consequently, at the early stage of solidification, the melt moves from the top region of the columnar grains toward the bottom, passing through the dendritic structures and forming two prominent symmetric vortices before flowing upward along the sample boundaries. Similar simulation results can also be found in previous literature [13]. As the solidification progresses, dendritic grains appear gradually, with copper accumulation in the interdendritic area, and the typical symmetric vortices disappear at the front of the solidification instead of irregular downward flow patterns observed in Figure 5b,c. Meanwhile, the maximum values of melt flow increase from 4.5 × 10^−5^ m/s at 80 s to 6.7 × 10^−5^ m/s at 120 s as the solidification progresses, which results from the downward effects of thermal-solutal buoyancy forces. The solidification sequence results show that the initial nuclei are pre-set at the center of the bottom boundary and start to grow as the temperature decreases under the liquidus line. Meanwhile, the copper is rejected into the melt and accumulates at the front of the dendritic grains. As solidification progresses, the dendritic grains continue to grow, and copper becomes increasingly concentrated in the interdendritic regions of the columnar grains due to the downward convective transport induced by thermal and solutal buoyancy forces. Meanwhile, the maximum copper concentration rises from 4.9 wt.% at 40 s to 12.0 wt.% at 120 s, further intensifying the melt flow. In addition, a relatively flat interface is observed during solidification, which is quite close to the experimental results [20], with a similar morphology of dendritic columnar grains.

**Figure 6:**
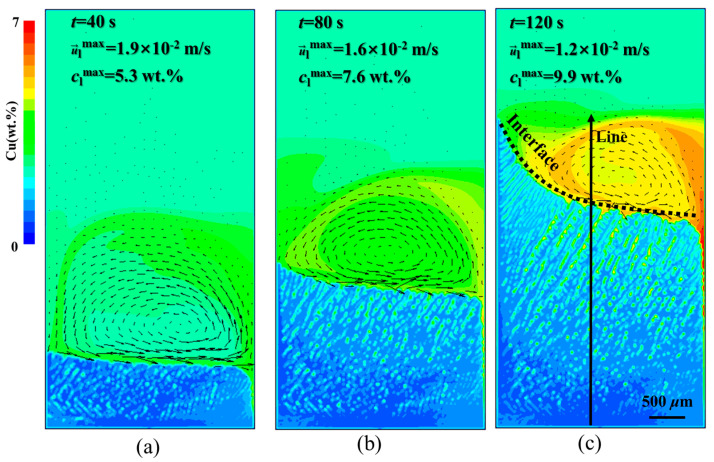
Typical characterization of columnar grain evolution, melt flow state, and solute distribution with steady magnetic field of 0.5 T: (**a**) 40 s, (**b**) 80 s, and (**c**) 120 s.

Additionally, for the case with a steady magnetic field of 0.5 T (Case 2), the simulated results of grain evolution, flow patterns, and the distribution of copper concentration are presented in Figure 6. In addition to the gravitationally driven buoyancy forces, two magnetic-field-related forces are considered: (a) the TEMF, which drives the melt to flow around the dendrites from left to right, and (b) the induced Lorentz force, which suppresses the flow’s intensity. The typical flow patterns in Case 2 are extremely different from those of Case 1. A qualitative comparison between these two cases shows that the liquid located at the front of the dendritic columnar grains flows from the left area to the right area and then flows upward close to the right-side wall. After that, the melt flows downward with the effects of gravity to the solidification interface area. Hence, a large-scale vortex of elliptical shape can be observed in the vicinity of the solidification front. The maximum magnitude of melt flow decreases from 1.9 × 10^−2^ m/s at 40 s to 1.2 × 10^−2^ m/s at 120 s as solidification progresses (with the same order of liquid velocity compared with previous research [13]), which may be attributed to the decreasing liquid domain during solidification. A quantitative comparison between the two cases reveals that the magnitude of the melt flow velocity is on the order of 10^−2^ m/s under a steady magnetic field, which is significantly larger than the 10^−5^ m/s observed without a magnetic field. This substantial increase demonstrates that the TEMF plays a dominant role in governing melt flow when a steady magnetic field is applied. Consequently, driven by this drastically altered flow state, the rejected copper is transported from the left region and forced to accumulate near the right-side wall. A distinct copper-rich accumulation area can be observed at the right side of the simulation domain during solidification. According to the phase diagram as shown in Figure 1, the liquidus line of the melt decreases with the increase in copper concentration. Hence, the accumulation of copper on the right side will suppress the dendritic grains’ growth, while the dendritic grains in the left area have a limited effect of copper-rich melt. Finally, the columnar grains grow fast in the left area with a lower copper concentration compared with that of the melt on the right side. A tilted interface is observed during solidification, and a similar morphology of dendritic columnar grains can also be observed in the experimental results [20].

**Figure 7:**
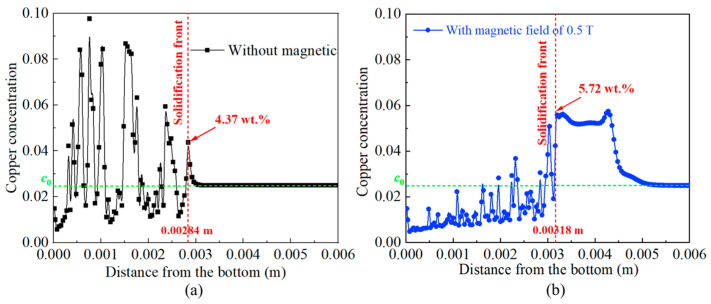
Quantitative comparisons of copper concentration profiles along the lines indicated in Figure 5c and Figure 6c (**a**) for Case 1 and (**b**) for Case 2.

Furthermore, the quantitative comparisons of the copper concentration profile, extracted along the reference lines shown in Figure 5c and Figure 6c, are presented in Figure 7. It is easy to conclude that the distance of the solidification front from the bottom wall is 0.00284 m for Case 1 with a copper concentration of 4.37 wt.% and 0.00318 m for Case 2 with a copper concentration of 5.72 wt.%, which quantitatively demonstrates that the copper-rich melt accumulates at the front of the solidification interface. The length of growth of 0.00318 m in Case 2 is greater than the 0.00284 m observed in Case 1. As noted in our previous research [14], this enhancement likely results from the accelerated transfer of copper out of the solidification cells and into the melt cells, driven by the TEMF. Consequently, the dendrites experience rapid growth, leading to an even higher accumulation of rejected copper within the melt at the solidification front.

### 3.3. Influence of Different Steady Magnetic Field Intensities

**Figure 8:**
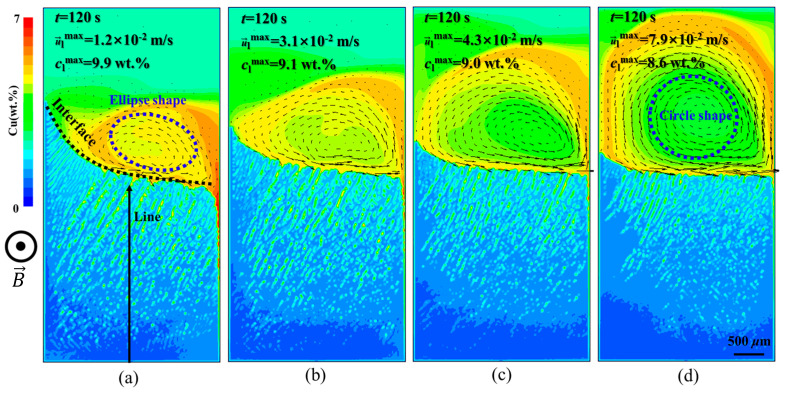
Typical characterization of columnar grain evolution, melt flow state, and solute distribution with different steady magnetic fields of (**a**) 0.5 T in Case 2, (**b**) 1.0 T in Case 3, (**c**) 2.0 T in Case 4, and (**d**) 4.0 T in Case 5 at 120 s.

**Figure 9:**
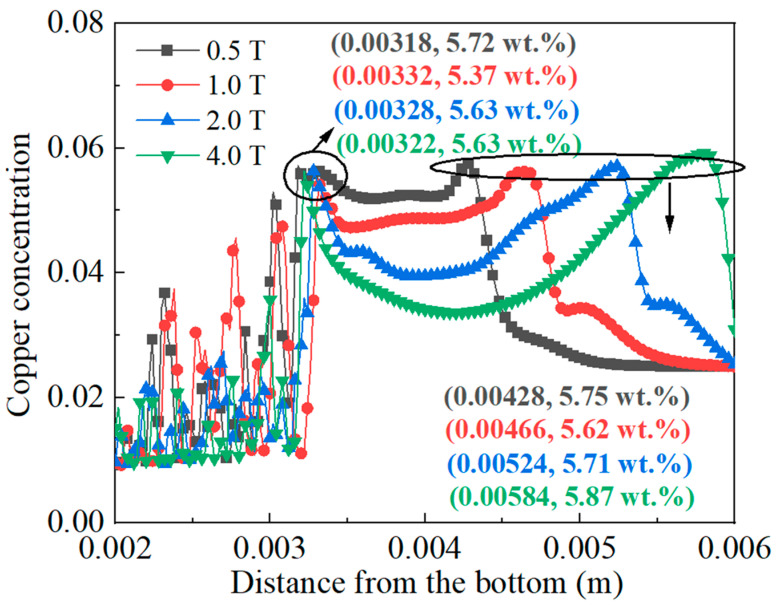
The copper concentration values along the line located at the center area of the simulation domain with different magnetic fields.

Both the simulated and experimental results show that the steady magnetic field has significant effects on the melt flow, leading to solute redistribution and dendritic grain growth. In this part, the simulation of different steady magnetic field intensities affecting the solidification behavior is presented. Four cases with a steady magnetic field of 0.5 T (Case 2), 1.0 T (Case 3), 2.0 T (Case 4), and 4.0 T (Case 5) are examined, and the morphology of columnar grains, melt flow state, and the distribution of copper concentration are presented in Figure 8. A distinct trend emerges, wherein increasing the magnetic field’s strength dramatically intensifies fluid flow within the melt. This observation aligns with theoretical expectations, as dictated by the Lorentz force expression of $\vec{J}\times \vec{B}.$ As the field’s strength rises, the maximum values of melt flow increase by nearly an order of magnitude, jumping from 1.2 × 10^−2^ m/s of 0.5 T to 7.9 × 10^−2^ m/s of 4.0 T. This intensification manifests as a large counterclockwise vortex located directly above the solidification interface. As shown in Figure 8a,d, the morphology of this vortex transitions from an ellipse to a circle as the magnetic field increases. This enhanced convection significantly alters the distribution of copper concentration and reduces solute accumulation in the interdendritic regions. Consequently, the maximum copper concentration drops from 9.9 wt.% in Case 2 (0.5 T) to 8.6 wt.% in Case 5 (4.0 T). Finally, the microstructure is strongly influenced by these hydrodynamic changes. In Case 2, a pronounced tilted solidification interface is observed. As the strength of the applied magnetic field, the vortex almost covers the whole solidification interface, as shown in Figure 8d, where the rejected copper flows from the right side to the left area along the circular vortex, significantly suppressing the solidification in the left area, leading to a relatively flat solidification interface compared with that for the other three cases. In order to further analyze the significant morphological changes of the solidification interface with different magnetic field strengths, the copper concentration values along the line (shown in Figure 8a, located at the center area of the simulation domain) are summarized in Figure 9. The distribution of copper concentration can be separated into two parts: (a) the concentration of dendrites and interdendritic melt below the solidification interface and (b) the concentration in the melt region. For the region below the solidification interface, the copper concentration fluctuates significantly for all cases, oscillating roughly between 1.0 wt.% and 5.0 wt.%. These fluctuations are attributed to the copper accumulation at the interdendritic area during the solidification. For the melt region above the solidification interface, two typical copper-rich peaks can be observed, one close to the solidification interface and another one far from it, depending on the strength of the magnetic field. These interesting copper distributions are attributed to the melt flow driven by the TEMF forming counterflow in the liquid region, as shown in previous results. The distances of these two copper-rich peaks are 0.00110 m for 0.5 T, 0.00134 m for 1.0 T, 0.00196 m for 2.0 T, and 0.00262 m for 4.0 T, and we can conclude that the distance increases gradually as a stronger magnetic field is applied. The Lorenz force of $\vec{J}\times \vec{B}$ shows that stronger magnetic fields lead to the increasing intensity of melt flow. The copper-rich melt flows along the anticlockwise vortex passing through the far melt and back to another side of the solidification interface. Meanwhile, copper-barren areas occur at the center of the vortices, leading to copper-rich peaks. All in all, the solidification interface becomes relatively flat with increasing magnetic field strengths, which results from the formation of a larger vortex and copper redistribution.

### 3.4. Grain Refinement

**Figure 10:**
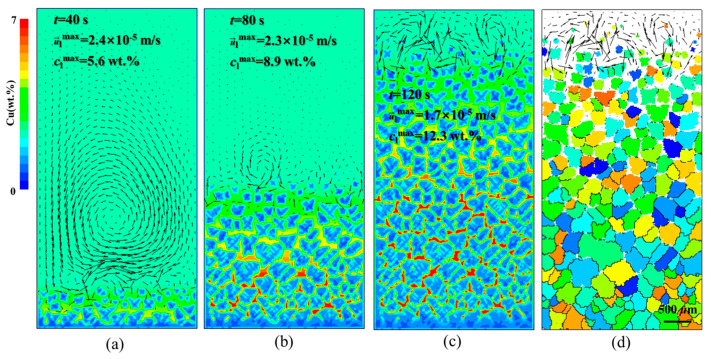
Typical evolution of equiaxed grains’ morphology, melt flow state, and solute distribution without a magnetic field: (**a**) 40 s, (**b**) 80 s, and (**c**) 120 s. (**d**) Microstructural morphology.

The typical evolution of equiaxed grains morphology, melt flow state, and the distribution of copper concentration without a magnetic field (Case 6) is presented in Figure 10. At the early stage of 40 s, the solid nucleus randomly forms, based on the nucleation model, and grows up gradually. A large anticlockwise vortex with a maximum average velocity of 2.4 × 10^−5^ m/s is observed far from the solidification interface, dominated by thermal and solutal buoyancy forces, while some irregular vortices occur at the interdendritic area. As solidification progresses, the accumulating equiaxed grains physically impede the fluid’s motion, forcing the vortex to shrink and migrate to the upper region of the domain, and the value of maximum melt flow decays to 1.7 × 10^−5^ m/s by 120 s. Meanwhile, the copper is rejected into the melt and increases gradually during solidification from 5.6 wt.% of 40 s to 12.3 wt.% 120 s. Furthermore, the corresponding microstructure presented in Figure 10d indicates that equiaxed grains form during solidification, which is consistent with similar experimental observations [22].

**Figure 11:**
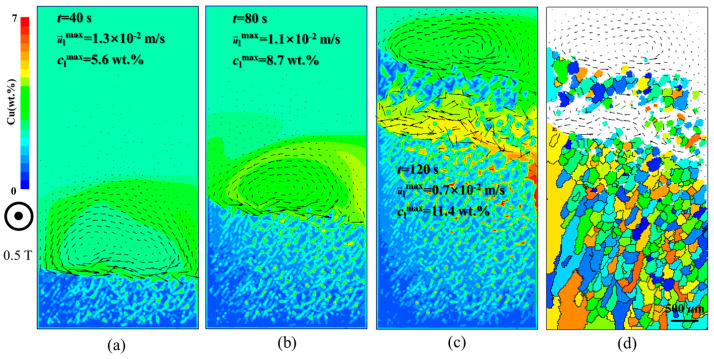
Typical evolutions of equiaxed grains’ morphology, melt flow state, and solute distribution with a steady magnetic field of 0.5 T: (**a**) 40 s, (**b**) 80 s, and (**c**) 120 s. (**d**) Microstructural morphology.

**Figure 12:**
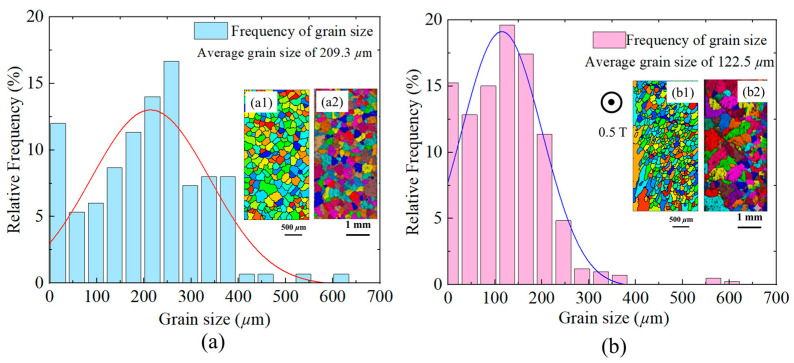
The final characteristics of the microstructure under different parameters: (**a**) without a magnetic field in Case 6; (**b**) under a magnetic field in Case 7. (**a1**,**b1**) simulated results, (**a2**,**b2**) experimental results.

Typical evolutions of equiaxed grains’ morphology, melt flow state, and solute distribution with a steady magnetic field of 0.5 T are presented in Figure 11. At the early stage of 40 s, the equiaxed grains nucleate and grow up under TEMF, leading to the liquid flowing from the left area to the right area, and a significant anticlockwise vortex of elliptical shape is observed in the liquid domain with a maximum average velocity of 1.3 × 10^−2^ m/s. As the solidification progresses, the vortex occurs at the solidification front, while the shrinking of the liquid domain makes the melt flow reduce to 0.7 × 10^−2^ m/s at 120 s. Meanwhile, a similar copper-rich distribution can be observed at the early stage compared with Case 2. However, as the copper accumulates at the front of the solidification interface, it suppresses the grain growth. Some equiaxed grains nucleate at the area far from the solidification interface, which should settle down in the experimental results [23], which could not be reproduced in current simulation due to the limit of the current model assuming the solid phase to be stationary. However, we can also achieve the mechanism of refinement under TEMF. The final characteristics of the microstructure under different parameters are presented in Figure 12. A purely equiaxed microstructure can be observed in Case 6 with an average grain size of 209.3 μm. The similar experimental morphology of the microstructure is also presented in Figure 12(a2) in the results [23]. However, the average grain size in Case 7 is 122.5 μm, with extremely grain refinement occurring compared with Case 6. Meanwhile, unlike the purely equiaxed microstructure seen in Case 6, two distinct types of microstructures can be observed in Figure 12(b1): (a) elongated equiaxed grains in the left area of the domain and (b) refined equiaxed grains in the right area of the domain. Similar microstructural distribution can also be observed in the experimental results [23].

## 4. Discussion

### 4.1. Mechanism of Grain Refinement

**Figure 13:**
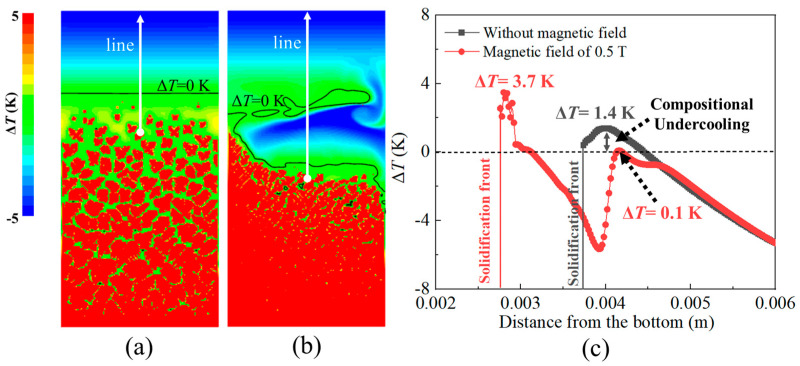
The characteristics of undercooling (Δ*T*) close to the solidification interface at 102.5 s: (**a**) without a magnetic field in Case 6; (**b**) under a magnetic field in Case 7. (**c**) Quantitative comparison of undercooling along the corresponding line.

In order to discuss the mechanism of refinement under a magnetic field, the undercooling (Δ*T*) for these cases close to the solidification interface at 102.5 s is presented in Figure 13. Meanwhile, the detailed distributions of undercooling along the corresponding line (marked in Figure 13a,b) are shown in Figure 13c. We also should emphasize that the average cooling rate in our study can be calculated explicitly by the equation $\dot{T}=G\cdot R$. In order to decrease the effects of the cooling rate on grain nucleation, Cases 6 and 7 are set with the same solidification processing parameters to compare the effects of the steady magnetic field. According to the model’s description, the undercooling is defined as $\Delta T=T_{f}+mc_{\mathrm{mix}}-T$, where T is the local temperature and $c_{\mathrm{mix}}$ is the average concentration of copper at the local cell.

For Case 6, the interface of Δ*T* = 0 is almost flat, which means the undercooling decreases gradually far away from the solidification front, and the maximum undercooling is 1.4 K, with a significant compositional undercooling area occurring close to the solidification interface. However, the undercooling distribution of Case 7 shows that it decreases far away from the solidification interface and then increases, forming a positive area before falling to a negative region again. Hence, a significant area with negative undercooling is located at the front of the solidification interface between two positive undercooling areas, which is mainly attributed to the distribution of copper concentration under the effect of TEMF on melt flow. As in the typical distribution of copper concentration in Figure 11b, two copper-rich peaks will be observed far from the solidification interface. For the copper-rich melt, it has a lower liquidus temperature, according to the phase diagram, and the undercooling decreases under the specific local temperature. The quantitative results show the first positive area with 3.7 K close to the solidification interface and another area with 0.1 K for Case 7, as shown in Figure 13c. As shown in Equation (8) in Section 2.6, an increase in ∆*T* will promote the nucleation of grains. For the quantitative comparison, Case 7 not only increases the magnitude of undercooling but also creates a new area for nucleation because of the copper concentration’s redistribution under the effect of TEMF. Finally, the microstructural refinement is observed in both the numerical and experimental results.

### 4.2. Prospects of the Current Simulation Framework

While the present numerical framework successfully captures the primary phenomena driving steady magnetic field-assisted solidification, several limitations inherent to its foundational assumptions must be noted. First, the reliance on constant thermophysical properties and the treatment of the solid phase as strictly stationary precludes the capture of localized dynamic phenomena, such as dendrite fragmentation, grain movement, and shrinkage-induced porosity, which can influence the final microstructure. Furthermore, because TEMF relies fundamentally on the disparity in Seebeck coefficients between the solid and liquid phases, the intensity of the proposed mechanism will vary across different alloy systems. Applying this framework to alloys with negligible thermoelectric disparities would yield a substantially different flow regime, requiring careful recalibration of the TEMF source terms. Finally, extrapolating this model to alternative magnetic field configurations, such as high-frequency alternating or traveling magnetic fields, would necessitate expanding the governing equations to account for transient induction effects, skin depth variations, and time-varying Lorentz forces. Despite these simplifications, the established mechanisms of thermal-solutal and TEMF-induced convection provide a robust foundational understanding that can be readily adapted to evaluate the solidification of other advanced structural alloys under varied electromagnetic processing conditions.

## 5. Conclusions

In this study, a cellular automation method coupled with a Eulerian multiphase approach was employed to numerically investigate the effects of varying steady magnetic fields on melt flow, solute distribution, grain evolution, and the final microstructure during the directional solidification of an Al-2.5 wt.% Cu alloy. The comprehensive model accounts for heat and solute transport, grain nucleation and growth, complex melt flows driven by thermal-solutal buoyancy, and TEMF and the induced Lorentz forces. The principal conclusions are summarized as follows.

(1) Under a steady magnetic field of 0.5 T, the melt flow keeps an order of 10^−2^ m/s, which leads to an elliptical copper-rich region forming in the vicinity of the solidification front. This localized solute redistribution significantly drives the formation of a tilted solid–liquid interface, a phenomenon well corroborated by experimental observations.
(2) As the magnetic field strength increases from 0.5 T to 4.0 T, the morphology of the copper-rich region at the solidification front transitions from elliptical to circular gradually. Notably, under a 4.0 T magnetic field, the tilted interface is effectively stabilized due to the strong suppression of grain growth.
(3) Significant grain refinement is achieved under a steady 0.5 T magnetic field with an average grain size of 122.5 μm, compared with 209.3 μm in the case without a magnetic field. This refinement is primarily attributed to an increase in the undercooling and the generation of new nucleation zones. This solute-driven mechanism is identified as the dominant factor for tailoring microstructures under steady magnetic fields.

## Figures and Tables

**Table 1:** Main conservation equations for each corresponding phase.

Description	Equations
**Mass conservations**	$\frac{\partial }{\partial t}\left(f_{l}\rho_{l}\right)+\nabla \cdot \left(f_{l}\rho_{l}\vec{u_{l}}\right)=-M_{\mathrm{ls}}$ $\;\frac{\partial }{\partial t}\left(f_{s}\rho_{s}\right)=M_{\mathrm{ls}}$
**Momentum conservations**	$\frac{\partial }{\partial t}\left(f_{l}\rho_{l}\vec{u_{l}}\right)+\nabla \cdot \left(f_{l}\rho_{l}\vec{u_{l}}\times \vec{u_{l}}\right)=-f_{l}\nabla P+\nabla \cdot \overset{̿}{\tau_{l}}-{\vec{U}}_{\mathrm{ls}}+\vec{J}\times \vec{B}+{\vec{f}}_{\rho }$
**Enthalpy conservations**	$\frac{\partial }{\partial t}\left(f_{l}\rho_{l}h_{l}\right)+\nabla \cdot \left(f_{l}\rho_{l}\vec{u_{l}}h_{l}\right)=\nabla \cdot (f_{l}k_{l}\text{∇}T_{l})-Q_{\mathrm{ls}}$ $\;\frac{\partial }{\partial t}\left(f_{s}\rho_{s}h_{s}\right)=\nabla \cdot (f_{s}k_{s}\text{∇}T_{s})+Q_{\mathrm{ls}}$
**Species conservations**	$\frac{\partial }{\partial t}\left(f_{l}\rho_{l}c_{l}\right)+\nabla \cdot \left(f_{l}\rho_{l}\vec{u_{l}}c_{l}\right)=\nabla (f_{l}\rho_{l}D_{l}\nabla c_{l})-C_{\mathrm{ls}}$ $\;\frac{\partial }{\partial t}\left(f_{s}\rho_{s}c_{s}\right)={\nabla (f_{s}\rho_{s}D_{s}\nabla c_{s})+C}_{\mathrm{ls}}$

**Table 2:** Physical properties of the Al-2.5 wt.% Cu alloy employed in all simulation cases.

Properties	Units	Quantity	Reference
**Initial concentration (*c*_0_)**	wt.%	2.5	-
**Density (*ρ*_l_/*ρ*_s_)**	kg·m^−3^	2780	[15]
**Equilibrium partition coefficient (*k*)**	1	0.17	[15]
**Liquidus slope (*m*)**	K·(wt.%)^−1^	−336	[15]
**Melting point at *c* = 0 (*T*_f_)**	K	933.6	[13]
**Thermal conductivity (***k*_l_/*k*_s_**)**	W·m^−1^·K^−1^	87	[15]
**Specific heat (*c*_p_)**	J·kg^−1^·K^−1^	1086	[15]
**Viscosity (μL)**	kg·m^−1^·s^−1^	0.0014	[15]
**Latent heat (**Δ*h*_f_**)**	J·kg^−1^	389,320	[15]
**Diffusion coefficient (liquid) (*D*_l_)**	m^2^·s^−1^	3.0 × 10^−9^	[13]
**Thermal expansion coefficient (*β*_T_)**	K^−1^	0.000117	[13]
**Solutal expansion coefficient (*β*_c_)**	wt.%^−1^	−1.0	[13]
**Electrical conductivity of liquid**	S/m	3.8 × 10^6^	[13]
**Electrical conductivity of solid**	S/m	1.3 × 10^7^	[12]
**Seebeck coefficient of liquid (S_l_)**	V/K	−2.25 × 10^−6^	[7]
**Seebeck coefficient of solid (Ss)**	V/K	−1.5 × 10^−6^	[7]

**Table 3:** Detailed processing parameters [20] used in all simulation cases.

	Magnetic Field (T)	Grain Morphology Assumption	Temperature Gradient (K/m)	Pulling Rate (m/s)
Case 1	No	Columnar grain	6000	50 × 10^−6^
Case 2	0.5	Columnar grain	6000	50 × 10^−6^
Case 3	1.0	Columnar grain	6000	50 × 10^−6^
Case 4	2.0	Columnar grain	6000	50 × 10^−6^
Case 5	4.0	Columnar grain	6000	50 × 10^−6^
Case 6	No	Equiaxed grain	6000	50 × 10^−6^
Case 7	0.5	Equiaxed grain	6000	50 × 10^−6^

**Table 4:** Numerical parameters used in all simulation cases.

Parameters	Units	Quantity
Time step	s	5 × 10^−3^
Mesh size	μm	20
Simulation domain	μm	3000 × 6000
Total mesh amount	-	45,000
Number of iterations for each time step	-	60
Convergence limit for each time step	-	10^−5^

## Data Availability

The original contributions presented in this study are included in the article. Further inquiries can be directed to the corresponding author.
